# Uridine diphosphate glucuronosyltransferase 1A1 gene polymorphisms and treatment outcomes in HIV and MTB coinfection in sub-Saharan Africa: a scoping review protocol

**DOI:** 10.1136/bmjopen-2025-102785

**Published:** 2026-06-08

**Authors:** Donald Vhanda, Cuthbert Musarurwa, Raylton P Chikwati, Masimba Muziringa, Collet Dandara, Justen Manasa, Joconiah Chirenda, Rooyen Tinago Mavenyengwa

**Affiliations:** 1 Department of Laboratory Diagnostic and Investigative Sciences, Faculty of Medicine and Health Sciences, University of Zimbabwe, Harare, Zimbabwe; 2 Department of Human, Biological and Translational Medical Sciences, School of Health Sciences and Veterinary Medicine, University of Namibia, Windhoek, Khomas Region, Namibia; 3 SAMRC/Wits Ageing African Adult Research Unit, Department of Paediatrics, School of Clinical Medicine, University of the Witwatersrand Johannesburg Faculty of Health Sciences, Johannesburg, Grand Prix, South Africa; 4 Library, University of Zimbabwe, Harare, Harare Province, Zimbabwe; 5 Department of Pathology and Institute of Infectious Disease and Molecular Medicine (IDM), Division of Human Genetics, University of Cape Town, Observatory, Cape Town, South Africa; 6 Department of Internal Medicine, University of Zimbabwe Faculty of Medicine and Health Sciences, Harare, Harare Province, Zimbabwe; 7 Biomedical Research and Training Institute, Harare, Harare Province, Zimbabwe; 8 Department of Global Public Health and Family Medicine, Faculty of Medicine and Health Sciences, University of Zimbabwe, Harare, Zimbabwe

**Keywords:** UGT1A1, genetic polymorphisms, HIV and TB, dolutegravir, Treatment outcomes

## Abstract

**Abstract:**

**Introduction:**

Uridine diphosphate glucuronosyltransferase 1A1 (UGT1A1) is closely associated with the management of HIV and tuberculosis (TB) coinfection because it modulates the metabolism of antiretroviral (ARV) drugs. The frequency of UGT1A1 polymorphisms varies widely among sub-Saharan Africans. However, studies examining the frequency of UGT1A1 polymorphisms and their impact on drug response profiles, accounting for environmental factors, drug–drug and gene–drug interactions and non-compliance remain sparse. Given that HIV and TB treatments often involve complex drug regimens with a high risk of interactions, understanding the role of UGT1A1 polymorphisms in these contexts is crucial. Therefore, this scoping review aims to map existing evidence, synthesise findings on how genetic polymorphisms in the UGT1A1 gene affect the metabolism of ARVs and antituberculosis drugs, and identify gaps in literature regarding their impacts on drug efficacy, toxicity and treatment outcomes in sub-Saharan Africa (SSA).

**Methods and analysis:**

The methodology for this scoping review will follow the guidelines outlined in the Joanna Briggs Institute Methodology Manual. Using the keywords, UGT1A1 polymorphism, HIV and TB coinfection, treatment outcomes and SSA, we will search for articles on PubMed/Medline, Cochrane Library, Embase, Web of Science and Scopus to obtain relevant articles published from January 2010 to April 2026. Two independent reviewers will screen and assess quality of titles and abstracts against the predefined inclusion and exclusion criteria and manage the data using Microsoft Excel. Conflicts will be resolved through discussion and where necessary a third reviewer will be consulted. Findings will be narratively synthesised across polymorphisms and treatment outcomes. The reviewers will meet and discuss the themes that will arise as well as the interpretation of the themes to minimise bias in the findings.

**Ethics and dissemination:**

The scoping review relies on publicly available published resources, exempting it from ethical review board oversight. The review findings will be shared in a peer-reviewed journal.

**STRENGTHS AND LIMITATIONS OF THIS STUDY**

- This review protocol describes a rigorous search strategy (Joanna Briggs Institute methodology) and the use of Preferred Reporting Items for Systematic Reviews and Meta-Analyses extension for Scoping Review (PRISMA-ScR) 2020 guideline for reporting on the findings of how genetic polymorphisms in the uridine diphosphate glucuronosyltransferase 1A1 gene affect the metabolism of antiretroviral (ARV) and antituberculosis treatment (ATT) drugs, and gaps in literature regarding these impacts in sub-Saharan Africa.
- This methodology is widely regarded as a robust and reliable source of evidence, providing valuable insights for researchers, policymakers and practitioners to inform decision-making, guide policy development and improve practice
- The search algorithm was developed in consultation with a subject expert librarian and customised for multiple databases and the grey literature, inclusive of peer-reviewed literature.
- The limitations of the proposed review will be the exclusion of non-English language studies and the exclusion of studies that do not focus on rifampicin-based ATT and non-dolutegravir-based ARV therapy.

## Introduction

 Globally***,*** sub-Saharan Africa (SSA) bears the largest burden of HIV infections (∼65%) and HIV-*Mycobacterium tuberculosis* (MTB) co-infections.^1 2^ HIV-MTB coinfections necessitate antiretroviral (ARV) and antituberculosis treatment (ATT) cotreatment.^3^ Comanagement with dolutegravir-based antiretroviral therapy (ART) and rifampicin-based ATT has been known to induce the enzyme uridine diphosphate glucuronosyltransferase 1A1 (UGT1A1) thus increasing its expression and activity, leading to increased metabolism of dolutegravir. Increased metabolism of dolutegravir may result in failed virological suppression, potentially leading to HIV drug resistance.^4^,^6^

The rate of UGT1A1 induction has shown individual variation, with UGT1A1*36 allele associated with increased enzyme activity, UGT1A1*1 allele linked with normal enzyme activity and UGT1A1*6, UGT1A1*28 alleles associated with diminished enzyme activity.^6^,^9^ Such patients might therefore require different tailoring of their dolutegravir treatment protocols to avoid underdosing or overdosing, thus potentiating the role of public precision medicine in infectious diseases.^10^

The UGT1A1 polymorphism frequencies vary widely among SSAs.^8^ Therefore, this scoping review aims to map existing evidence, synthesise findings on how genetic polymorphisms in the UGT1A1 gene affect the metabolism of ARV and ATT drugs, and identify gaps in literature regarding their impacts on drug efficacy, toxicity and treatment outcomes in SSA.^5 11^ Examining the impact of UGT1A1 genetic polymorphisms on HIV and tuberculosis (TB) treatment outcomes is vital to inform quality care in HIV-MTB coinfections.

The researchers chose a scoping review approach because it provides an expanse and overview of the existing evidence on the UGT1A1 genetic polymorphisms and management of HIV-MTB coinfections.^12^ The study will focus on how genetics influences drug–drug interactions in patients being treated for the two conditions concurrently. Our findings will contribute to a better understanding of the interactions and associated treatment outcomes, thus informing policy, practice and possible research gaps. Healthcare professionals, researchers and policymakers will benefit from the findings in tailoring targeted interventions for respective patient groups.

To our knowledge, the few scoping reviews have gaps in knowledge related to the role of genetic variants and drug–drug–gene interactions on the treatment outcomes in African HIV and MTB coinfected populations. Many studies have focused on the pharmacokinetics of rifampicin induction on DTG.^913^,^15^ A few studies have investigated the influence of genetics on selected treatment outcomes, but none have been done in SSA, where the pharmacogenetic variation is highly diverse. The studies primarily concentrated on specific outcomes, resulting in notable gaps in evidence, knowledge and empirical understanding, as well as unaddressed population-level concerns. There is also limited data on the efficacy of DTG-based ART combinations when coadministered with rifampicin-based anti-TB drugs in real-life settings in SSA.

### Objectives

The main objective of this scoping review is to summarise the available evidence on the effect of UGT1A1 genetic polymorphisms on drug concentrations and treatment outcomes in HIV and MTB coinfected people on dolutegravir and rifampicin cotreatment in SSA. Specifically, for individuals taking dolutegravir and rifampicin concurrently in SSA. The review intends to uncover information on the:

1. Common genetic polymorphisms of the UGT1A1 gene in SSA.
2. Effect of UGT1A1 polymorphisms on dolutegravir concentration and treatment outcomes.
3. Risk factors associated with poor treatment outcomes in HIV and MTB coinfected populations.

## Methods and analysis

The methodology for this scoping review will follow the guidelines from the Joanna Briggs Institute (JBI) Methodology Manual for scoping reviews with the assistance of a medical librarian using Medical Subject Heading (MeSH) and individual keywords illustrated in table 1.^16^ This scoping review will follow the Preferred Reporting Items for Systematic Reviews and Meta-Analyses extension for Scoping Review (PRISMA-ScR) 2020 guidelines to increase transparency, accuracy and completeness of the findings.^17^ This scoping review protocol was registered on the Open Science Framework. The electronic databases that will be searched will include: PubMed/Medline, Cochrane Library, Embase, Web of Science and Scopus. Additional relevant studies that form part of the grey literature sources such as reports and theses will also be reviewed. The reference lists of included studies, reviews and guidelines will be searched manually to identify other relevant publications. To optimise the search for published literature, we will also search the internet for additional reviews as well as national guidelines worldwide. The study is planned to be conducted from 25 May 2026 to 30 September 2026. The following stages will be followed;

**Table 1:** Search strategy

Keyword	Alternative terms
UGT1A1 Polymorphism	Uridine Diphosphate Glucuronosyltransferase 1A1 polymorphism OR Uridine Diphosphate Glucuronosyltransferase 1A1 OR UGT1A1 OR UGT1A1 polymorphisms OR UGT1A1 variants OR UGT1A1 gene OR UGT1A1 gene mutation OR UGT1A1 allelic variation OR UGT1A1 genetic variant OR UGT1A1 genetic variation OR UGT1A1 genetic polymorphism OR UGT1A1 gene polymorphism OR UGT1A1 genotype OR UGT1A1 enzyme polymorphism OR UDP-glucuronosyltransferase polymorphism OR UGT1A1 single nucleotide polymorphisms OR UGT1A1 SNPs OR UGT1A1 molecular variations OR UGT1A1 genetic diversity OR UGT1A1 genetic heterogeneity
HIV and MTB Coinfection	HIV/MTB coinfection OR HIV and M.tuberculosis dual infection OR HIV-associated TB OR HIV and MTB comorbidity OR HIV/MTB comorbidity OR co-morbid HIV-MTB OR Tuberculosis in HIV patients OR MTB in HIV-infected individuals OR HIV and TB co-morbidity OR Tuberculosis and HIV infection OR MTB and HIV-coinfected OR TB/HIV epidemic OR HIV-MTB co-infection OR MTB-HIV coinfection OR Mycobacterium tuberculosis in HIV patients OR HIV-positive TB patients OR HIV/AIDS and tuberculosis OR HIV and MTB dual infection OR HIV associated TB OR concurrent HIV and MTB infection OR TB-HIV syndemic OR dual epidemic HIV and TB
Treatment Outcomes	Treatment Outcomes OR clinical outcomes OR patient outcomes OR health outcomes treatment response OR patient response OR patient response profiles OR treatment success rate OR efficacy OR adverse effects OR adverse drug reactions OR side effects OR susceptibility OR survival OR treatment profiles OR pharmacokinetics OR pharmacodynamics OR dolutegravir plasma concentrations OR DTG plasma concentrations OR dolutegravir concentrations OR DTG concentrations OR viral load OR viral suppression OR HIV drug resistance OR HIVDR or Neuropsychiatric disorders OR neuropsychiatric events OR liver function OR kidney function OR CD4 levels OR immune status OR weight gain
Filters- Sub-Saharan Africa	Sub-Saharan Africa OR Angola OR Benin OR Botswana OR Burkina Faso OR Burundi OR Cabo Verde OR Cameroon OR Central African Republic OR Chad OR Comoros OR Democratic Republic of Congo or Djibouti OR Equatorial Guinea OR Eritrea OR Eswatini OR Ethiopia OR Gabon OR Gambia OR Ghana OR Guinea OR Guinea-Bissau OR Ivory Coast OR Kenya OR Lesotho OR Liberia OR Madagascar OR Malawi OR Mali OR Mauritania OR Mauritius OR Mozambique OR Namibia OR Niger OR Nigeria OR Rwanda OR Sao Tome and Principe OR Senegal OR Seychelles OR Sierra Leone OR Somalia OR South Africa OR South Sudan OR Sudan OR Republic of Congo OR Tanzania OR Togo OR Uganda OR Zambia OR Zimbabwe

MTB, *Mycobacterium tuberculosis*; UGT1A1, uridine diphosphate glucuronosyltransferase 1A1.

### Stage 1: Identifying the research question

This scoping review aims to synthesise the available evidence on the effect of UGT1A1 genetic polymorphisms on treatment outcomes in patients coinfected with HIV and TB, and taking dolutegravir and rifampicin concurrently in SSA. The overall research question is ‘What is the available evidence on the effect of UGT1A1 polymorphisms on dolutegravir concentrations and treatment outcomes in HIV and MTB coinfection in SSA?’ Subquestions that will be answered include:

- What are the common UGT1A1 polymorphisms and their frequencies in SSA?
- What is the nature and extent of evidence on the impact of UGT1A1 polymorphisms on DTG pharmacokinetics in SSA settings?
- What patient-related, disease-related and treatment-related factors are associated with suboptimal dolutegravir treatment outcomes, including reduced efficacy, increased toxicity or poor adherence, in individuals coinfected with HIV and MTB in SSA?

### Stage 2: Identifying relevant studies

The researchers will work with a librarian to search for peer-reviewed journals and retrieve relevant studies. Only articles published in English will be reviewed. The electronic databases that will be searched will include: PubMed/Medline, Embase, Web of Science and Scopus. A combination of keywords and MeSH terms will be used to capture all the relevant studies on UGT1A1 polymorphisms, HIV and MTB coinfection, treatment outcomes in SSA. The search strategy will use Boolean operators (AND, OR) to maximise inclusion. Table 1 shows the search strategy for the scoping review. Online supplemental file 1 includes the search query for the databases, including all keywords and synonyms.

Additional relevant studies that form part of the grey literature sources such as reports and theses will be reviewed. Google Scholar will be used as a supplementary source, and reference lists of included studies will be screened for additional studies. The reference lists of key articles and reviews on UGT1A1, HIV and TB in SSA will be hand-searched to identify any relevant articles not captured by the database searches.

Table 2 summarises the inclusion and exclusion criteria. The screening process will be managed and documented using Microsoft Excel. Two independent reviewers will screen titles and abstracts against the predefined inclusion and exclusion criteria. Two reviewers will independently scrutinise the titles and abstracts of identified articles for eligibility. The screening will adopt and use standardised codes such as: Include, Exclude and Uncertain. Conflicts will be resolved through discussion, and where necessary, a third reviewer will be consulted. Full-text screening will follow the same procedure to ensure methodological rigour and transparency.

**Table 2:** Inclusion and exclusion criteria

Inclusion	Exclusion
**Study types:** observational studies (eg, cohort, case–control, cross-sectional studies), randomised controlled trials, non-randomised interventional studies (eg, prospective studies), systematic reviews and meta-analyses of studies related to UGT1A1 polymorphisms and treatment outcomes in HIV/MTB coinfection (used for context but not primary data), qualitative studies (if they explore patient perspectives on UGT1A1-related treatment outcomes in HIV/TB coinfection).	Animal studies or in vitro studies; Case reports or small case series (unless they present highly relevant or unique data not covered in larger studies); Studies focusing on diseases other than HIV or TB concerning UGT1A1 polymorphisms, Studies without a focus on SSA.
**Participants:** both adult and paediatric SSA populations will be included, provided the studies focus on HIV/MTB coinfection. Studies examining UGT1A1 polymorphisms in these populations. Studies that investigate the treatment outcomes of people living with HIV and TB on dolutegravir and rifampicin-based cotreatments.	Studies involving populations without confirmed HIV/MTB coinfection. Studies conducted outside SSA. Populations with no access to dolutegravir and rifampicin cotreatment, or those with conditions that would interfere with the treatment (eg, severe comorbidities not related to HIV/TB).
**UGT1A1 polymorphisms** Studies that investigate specific UGT1A1 gene polymorphisms (eg, UGT1A1*28, UGT1A1*6) UGT1A1*36, UGT1A1*37 and their effect in HIV/TB coinfection in SSA. Studies examining genetic variants of UGT1A1 and their role in the metabolism of dolutegravir-based ARVs and/or rifampicin-based anti-TB drugs.	Studies focused on other genetic polymorphisms unrelated to UGT1A1. Studies examining other metabolic pathways or enzymes that do not focus on UGT1A1.
**Treatment Outcomes** Studies that report on the efficacy, adverse effects or treatment outcomes of dolutegravir-based ARVs and rifampicin-based anti-TB therapies. Studies that report on survival rates, drug resistance and drug metabolism of dolutegravir-based ARVs and rifampicin-based anti-TB therapies. Studies that provide data on clinical or laboratory outcomes (eg, viral load suppression, TB clearance, side effects, adverse events, weight gain). Studies assessing the effect of UGT1A1 polymorphisms on dolutegravir plasma concentrations. Studies assessing the risk factors associated with poor treatment outcomes in SSA.	Studies that do not report on HIV and TB treatment outcomes (eg, studies focused only on basic science or diagnostics). Studies reporting only on general HIV or TB treatment without a connection to UGT1A1 polymorphisms. Studies not including data on the efficacy of treatment in relation to genetic variants.
Articles published in the English language.	Articles published in other languages.
Studies done in SSA.	Studies done outside SSA.
Articles published since between January 2010 and April 2026.	Articles published before 2010.

ARVs, antiretroviral drugs; MTB, *Mycobacterium tuberculosis*; SSA, sub-Saharan Africa; TB, tuberculosis; UGT1A1, uridine diphosphate glucuronosyltransferase 1A1.

### Stage 3: Study selection

**Figure 1:**
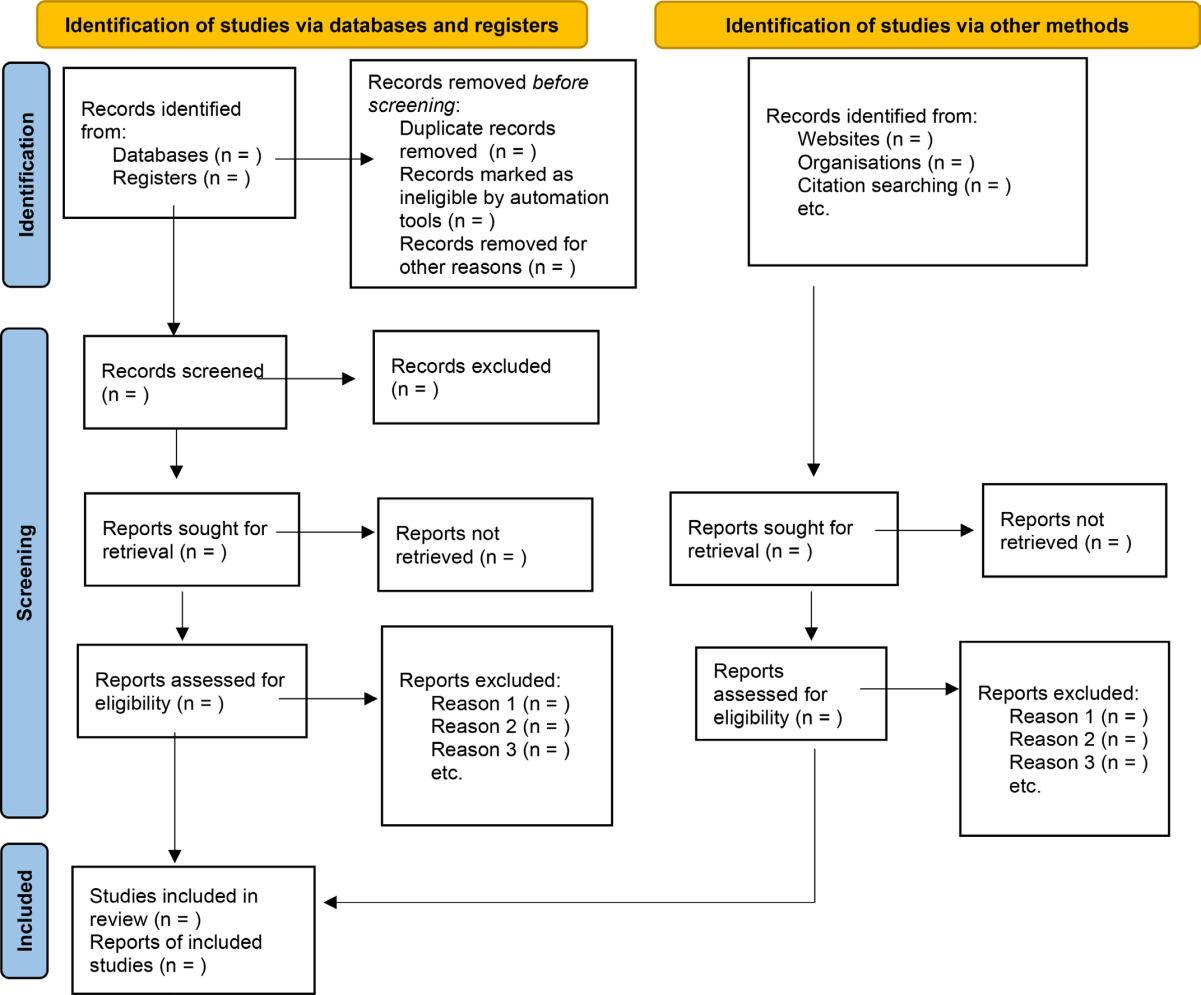
PRISMA 2020 flow diagram for systematic reviews which included searches for databases, registers and other sources. PRISMA, Preferred Reporting Items for Systematic Reviews and Meta-Analyses.

All articles identified from the databases will be exported to the Zotero reference manager, and duplicates will be removed. Deduplication will be conducted within Zotero using its automated duplicate detection function, that identifies records based on key bibliographic fields such as title, author(s) and publication year. Following automated removal, a manual review will be undertaken to ensure that any remaining duplicates not captured by the software are identified and removed, thereby enhancing the accuracy of the dataset. The final results will be profiled using the PRISMA-ScR workflow in figure 1.^18^ The PRISMA-ScR flow diagram provides a visual summary of the screening and selection of articles in a transparent and systematic approach. The reviewers will use Microsoft Excel sheets for the screening purpose. The spread sheets will have the following headings: article identification number, title, abstract, year of publication, journal name and URL.

## Charting the data extraction

A data extraction form will be developed to extract relevant data from the included studies. The data extraction form will be pilot-tested on a sample of five studies and appropriate amendments will be made to ensure its validity and reliability. An initial limited search will be performed in key databases such as PubMed to identify relevant articles and to analyse key terms and MeSH. These terms will then be used to optimise the full search strategy across all the selected databases. The pilot phase will also help ensure sensitivity and specificity of the search in capturing all relevant studies aligned to the review objectives. The data to be extracted will include studies investigating the UGT1A1 polymorphisms prevalent in SSA; their effect on treatment outcomes in patients coinfected with HIV and MTB and the risk factors associated with poor treatment outcomes. These will include observational studies (cohort studies, case–control studies and cross-sectional studies) and randomised controlled trials. The charting will also capture data on patients with HIV and MTB coinfection on dolutegravir and rifampicin cotreatment. The charting will also extract data on primary and secondary outcomes; treatment outcomes, dolutegravir plasma concentrations, treatment efficacy, adverse drug reactions, viral suppression, neuropsychiatric disorders, HIV drug resistance, liver and kidney function. Other study characteristics such as the first author, year of study and publication, study design, geographical location sample size, methodology, primary and secondary outcomes will also be collected.

## Collating, summarising and reporting the results

At this stage, the findings from the descriptive information will be presented. The retrieved articles will be appraised using JBI critical appraisal tools to assess the quality of the articles.^19^ This will ensure the utility of the disseminated results for policymakers, practice and also to inform future research in the pharmacogenomics of infectious diseases. Synthesis of the results will be done for articles appraised by summarising the charting results in relation to the review findings. The reviewers will meet and discuss the themes that will arise as well as the interpretation of the themes to minimise bias in the findings. In addition, they will identify and report on the gaps in the field of pharmacogenomics of HIV and TB as well as come up with recommendations for policy and practice. The reporting of the review will be done according to the PRISMA-ScR checklist.^19^

### Patient and public involvement

There will be no direct patient and public involvement in the design of the study as only published research articles will be used. However, findings from the scoping review will be shared with relevant stakeholders and published in a peer-reviewed journal.

### Ethics and dissemination

The scoping review relies on publicly available published resources, exempting it from ethical review board oversight. The review findings will be shared in a peer-reviewed journal.

### Implications

The scoping review will collate available evidence on UGT1A1 polymorphisms in HIV-TB cotreatment in SSA. This approach will therefore summarise the current evidence base and highlight emerging insights and research gaps. The scoping review has the potential to inform public health strategies through genetic screening and promoting precision medicine, thus improving patient outcomes by reducing drug interactions and adverse reactions. Furthermore, we will use this scoping review to inform the design of an observational study in Zimbabwe, a country in SSA with a high burden of HIV-MTB coinfections of approximately 54%. Policymakers, researchers and healthcare professionals will benefit from the findings in developing targeted interventions to address the treatment optimisation modalities in HIV and TB coinfected groups in SSA.

## Supplementary material

10.1136/bmjopen-2025-102785 online supplemental file 1
