# Supplementary material for: Uridine diphosphate glucuronosyltransferase 1A1 gene polymorphisms and treatment outcomes in HIV and MTB coinfection in sub-Saharan Africa: a scoping review protocol
Source: BMJ Open. 2026 Jun 8;16(6):e102785. doi: 10.1136/bmjopen-2025-102785 (PMC13250173; doi:10.1136/bmjopen-2025-102785)
Supplement: online supplemental file 1 [file bmjopen-16-6-s001.docx]

# Title

Uridine diphosphate glucuronosyltransferase 1A1 (UGT1A1) gene polymorphisms and treatment outcomes in HIV and TB coinfection in sub-Saharan Africa

# 1. Conceptual Framework (PCC)

| Population | HIV/TB coinfected patients |
| --- | --- |
| Concept | UGT1A1 polymorphisms (pharmacogenetics) |
| Context | Sub-Saharan Africa |

# 2. Key Search Concepts

| Gene/Pharmacogenetics | UGT1A1, polymorphism, variants, SNPs, pharmacogenetics |
| --- | --- |
| Disease | HIV, AIDS, Tuberculosis, TB, coinfection |
| Outcomes | treatment outcomes, treatment profiles, clinical outcomes, treatment success rate, toxicity, efficacy, dolutegravir concentrations, weight gain, neuropsychiatric events, neuropsychiatric disorders kidney function, kidney failure, liver function, liver failure, viral suppression, viral load, HIV drug resistance, adverse events, adverse drug reactions |
| Geography | Sub-Saharan Africa and individual countries |
|  |  |

# 3. PubMed- Core Boolean Search String

| UGT1A1 Polymorphism | "UGT1A1"[Mesh] OR UGT1A1[tiab] OR "uridine diphosphate glucuronosyltransferase 1A1"[tiab]  OR "UGT1A1 polymorphism*"[tiab] OR "UGT1A1 variant*"[tiab] OR "UGT1A1 sequence*"[tiab] OR OR pharmacogenetic*[tiab] OR pharmacogenomic*[tiab] OR UGT1A1 SNP* [tiab] OR “UGT1A1 gene” [tiab] OR “UDP-glucuronosyltransferase polymorphism” [tiab] OR “UGT1A1 genetic diversity” [tiab] OR “UGT1A1 genetic heterogeneity” [tiab] OR “UGT1A1 allelic variation” [tiab] OR “UGT1A1 enzyme polymorphism” [tiab] |
| --- | --- |
| HIV/TB Co-Infection | ("HIV"[Mesh] OR "HIV Infections"[Mesh] OR HIV[tiab] OR "human immunodeficiency virus"[tiab] OR AIDS[tiab]) **AND** ("Tuberculosis"[Mesh] OR tuberculosis[tiab] OR TB[tiab] OR "Mycobacterium tuberculosis"[tiab]) **AND** ("Coinfection"[Mesh] OR coinfection[tiab] OR "co-infection"[tiab] OR "HIV/TB"[tiab] OR "TB/HIV"[tiab] OR dual infection[tiab] OR concurrent infection[tiab] OR syndemic[tiab])  **AND**  ("people living with HIV and TB"[Mesh] |
| Treatment Outcomes | ("Treatment Outcome"[Mesh] OR "therapy outcome"[tiab] OR "treatment outcome*"[tiab] OR "clinical outcome*"[tiab]  OR "treatment response"[tiab] OR "response to treatment"[tiab] OR "treatment success"[tiab] OR "treatment failure"[tiab] OR "therapeutic outcome*"[tiab] OR "outcome assessment"[tiab]) "viral suppression"[tiab] OR "neuropsychiatric events"[tiab] OR "neuropsychiatric disorders*"[tiab] OR "neuropsychiatric adverse events*"[tiab] OR "dolutegravir concentrations"[tiab] OR "dolutegravir plasma concentrations*"[tiab] OR "kidney function"[tiab] OR "kidney profile*"[tiab] OR "kidney failure*"[tiab] OR "liver function"[tiab] OR "kidney assessment"[tiab] OR "liver failure*"[tiab] OR "liver assessment"[tiab] OR "weight gain"[tiab] OR "viral suppression"[tiab] OR "viral load"[tiab] OR "HIV drug resistance"[tiab] OR "adverse events"[tiab] OR "adverse drug reactions"[tiab] |

**4. Embase (Elsevier) -** Using EMTREE terms

|  | ('ugt1a1'/exp OR ugt1a1:ti,ab OR 'pharmacogenetics'/exp OR pharmacogenetic*:ti,ab) |
| --- | --- |
| **AND** | ('human immunodeficiency virus infection'/exp OR hiv:ti,ab) |
| **AND** | ('tuberculosis'/exp OR tuberculosis:ti,ab OR tb:ti,ab) |
| **AND** | ('coinfection'/exp OR coinfection*:ti,ab) |
| **AND** | ('treatment outcome'/exp OR 'drug toxicity'/exp  OR 'drug response':ti,ab OR toxicity:ti,ab OR adverse events':ti,ab OR 'viral suppression':ti,ab OR 'viral load':ti,ab OR 'neuropsychiatric events:ti,ab OR 'weight gain':ti,ab OR 'kidney function':ti,ab OR 'liver function':ti,ab OR 'dolutegravir plasma concentrations':ti,ab) |
| **AND** | ('sub-saharan africa'/exp OR 'africa south of the sahara':ti,ab  OR zimbabwe:ti,ab OR 'south africa':ti,ab OR ‘Angola’ ti,ab OR ‘Benin’ ti,ab OR ‘Botswana’ ti,ab OR ‘Burkina Faso’ ti,ab OR ‘Burundi’ ti,ab OR ‘Cabo Verde’ ti,ab OR ‘Cameroon’ ti,ab OR ‘Central African Republic’ ti,ab OR ‘Chad’ ti,ab OR ‘Comoros’ ti,ab OR ‘Democratic Republic of Congo’ ti,ab or ‘Djibouti’ ti,ab OR ‘Equatorial Guinea’ ti,ab OR ‘Eritrea’ ti,ab OR ‘Eswatini’ ti,ab OR ‘Ethiopia’ ti,ab OR ‘Gabon’ ti,ab OR ‘Gambia’ ti,ab OR ‘Ghana’ ti,ab OR ‘Guinea’ ti,ab OR ‘Guinea-Bissau’ ti,ab OR ‘Ivory Coast’ ti,ab OR ‘Kenya’ ti,ab OR ‘Lesotho’ ti,ab OR ‘Liberia’ ti,ab OR ‘Madagascar’ ti,ab OR ‘Malawi’ ti,ab OR ‘Mali’ ti,ab OR ‘Mauritania’ ti,ab OR ‘Mauritius’ ti,ab OR ‘Mozambique’ ti,ab OR ‘Namibia’ ti,ab OR ‘Niger’ ti,ab OR ‘Nigeria’ ti,ab OR ‘Rwanda’ ti,ab OR ‘Sao Tome and Principe’ ti,ab OR ‘Senegal’ ti,ab OR ‘Seychelles’ ti,ab OR ‘Sierra Leone’ ti,ab OR ‘Somalia’ ti,ab OR ‘South Africa’ ti,ab OR ‘South Sudan’ ti,ab OR ‘Sudan’ ti,ab OR ‘Republic of Congo’ ti,ab OR ‘Tanzania’ ti,ab OR ‘Togo’ ti,ab OR ‘Uganda’ ti,ab OR ‘Zambia’ ti,ab OR ‘Zimbabwe’ ti,ab). |

**5. Scopus/Web of Science**

|  | TITLE-ABS-KEY (UGT1A1 OR "uridine diphosphate glucuronosyltransferase 1A1"  OR pharmacogenetic* OR polymorphism*) |
| --- | --- |
| **AND** | TITLE-ABS-KEY (HIV OR AIDS) |
| **AND** | TITLE-ABS-KEY (tuberculosis OR TB) |
| **AND** | TITLE-ABS-KEY (“coinfection” OR “co-infection” OR comorbidity OR "co-morbidity" OR "dual infection" OR "concurrent infection" OR syndemic)) |
| **AND** | TITLE-ABS-KEY ("treatment outcome*" OR toxicity OR efficacy  OR "drug response" OR “adverse events”) |
| **AND** | TITLE-ABS-KEY ("sub-Saharan Africa" OR "Southern Africa" OR Angola OR Benin OR Botswana OR Burkina Faso OR Burundi OR Cabo Verde OR Cameroon OR Central African Republic OR Chad OR Comoros OR Democratic Republic of Congo or Djibouti OR Equatorial Guinea OR Eritrea OR Eswatini OR Ethiopia OR Gabon OR Gambia OR Ghana OR Guinea OR Guinea-Bissau OR Ivory Coast OR Kenya OR Lesotho OR Liberia OR Madagascar OR Malawi OR Mali OR Mauritania OR Mauritius OR Mozambique OR Namibia OR Niger OR Nigeria OR Rwanda OR Sao Tome and Principe OR Senegal OR Seychelles OR Sierra Leone OR Somalia OR South Africa OR South Sudan OR Sudan OR Republic of Congo OR Tanzania OR Togo OR Uganda OR Zambia OR Zimbabwe) |
|  |  |

**6. Cochrane Library**

|  | (UGT1A1 OR pharmacogenetic* OR polymorphism*) |
| --- | --- |
| **AND** | (HIV OR Human immunodeficiency virus OR AIDS OR Acquired Immunodeficiency syndrome) |
| **AND** | (tuberculosis OR TB) |
| **AND** | (coinfection OR co-infection OR dual infection OR concurrent infection OR syndemic infection) |
| **AND** | Treatment Outcomes OR clinical outcomes OR patient outcomes OR health outcomes treatment response OR patient response OR patient response profiles OR treatment success rate OR efficacy OR adverse effects OR adverse drug reactions OR side effects OR susceptibility OR survival OR treatment profiles OR pharmacokinetics OR pharmacodynamics OR dolutegravir plasma concentrations OR DTG plasma concentrations OR dolutegravir concentrations OR DTG concentrations OR viral load OR viral suppression OR HIV drug resistance OR HIVDR or Neuropsychiatric disorders OR neuropsychiatric events OR liver function OR kidney function OR CD4 levels OR immune status OR weight gain |
| **AND** | Sub-Saharan Africa OR Angola OR Benin OR Botswana OR Burkina Faso OR Burundi OR Cabo Verde OR Cameroon OR Central African Republic OR Chad OR Comoros OR Democratic Republic of Congo or Djibouti OR Equatorial Guinea OR Eritrea OR Eswatini OR Ethiopia OR Gabon OR Gambia OR Ghana OR Guinea OR Guinea-Bissau OR Ivory Coast OR Kenya OR Lesotho OR Liberia OR Madagascar OR Malawi OR Mali OR Mauritania OR Mauritius OR Mozambique OR Namibia OR Niger OR Nigeria OR Rwanda OR Sao Tome and Principe OR Senegal OR Seychelles OR Sierra Leone OR Somalia OR South Africa OR South Sudan OR Sudan OR Republic of Congo OR Tanzania OR Togo OR Uganda OR Zambia OR Zimbabwe |

# 7. Grey Literature Strategy

Include:

1. Google Scholar
2. African Journals Online (AJOL)
3. Institutional repositories (e.g., University theses)

Search example:

*“UGT1A1 HIV tuberculosis Africa pharmacogenetics and treatment outcomes”*

# 8. Additional Search Techniques

Citation chaining and manual journal searches

# 9. Limits

Language: English; Humans; Articles published between January 2010 to April 2026.
