# Supplementary material for: Uridine diphosphate glucuronosyltransferase 1A1 gene polymorphisms and treatment outcomes in HIV and MTB coinfection in sub-Saharan Africa: a scoping review protocol
Source: BMJ Open. 2026 Jun 8;16(6):e102785. doi: 10.1136/bmjopen-2025-102785 (PMC13250173; doi:10.1136/bmjopen-2025-102785)
Supplement: Supplementary file 2 [file bmjopen-2025-102785.reviewer_comments.pdf]

PEER REVIEW HISTORY

BMJ Open publishes all reviews undertaken for accepted manuscripts. Reviewers are asked to complete a checklist review form and are provided with free text boxes to elaborate on their assessment. These free text comments are reproduced below.

ARTICLE DETAILS

Title (Provisional)

Uridine diphosphate glucuronosyl transferase 1A1 gene polymorphisms and treatment outcomes in HIV and MTB coinfection in sub-Saharan Africa: A scoping review protocol

Authors

Vhanda, Donald; Musarurwa, Cuthbert; Chikwati, Raylton P; Muziringa, Masimba; Dandara, Collet; Manasa, Justen; Chirenda, Joconiah; Mavenyengwa, Rooyen T

VERSION 1 - REVIEW

Reviewer 1
Name Jiang, Qin
Affiliation Sichuan Provincial People's Hospital: Sichuan Academy of Medical Sciences and Sichuan People's Hospital
Date 21-Feb-2026
COI None

The authors provided many old references, many of them is dated from year 2020. Only one item was since 2020. Thus, the scientific value of this scoping review was restricted by timeliness.

Reviewer 2
Name Moyo-Chilufya , Maureen
Affiliation Faculty of Health Science University of Pretoria, School Of Health Systems and Public Health
Date 07-Apr-2026
COI None

Thank you for the opportunity to review the article titled, **“Uridine diphosphate glucuronosyl transferase 1A1 gene polymorphisms and treatment outcomes in HIV and MTB coinfection in sub-Saharan Africa: A scoping review protocol”** by Vhanda et al 2026

It is an important topic in the context of Sub -Saharan Africa, which has the highest burden of HIV, globally.

Abstract

Introduction: should begin with an introductory statement that explains the association of UGT1A1 with HIV/TB co-infection

Line 32 – 35: The aim does not state anything about UGT1A1. The authors should include the aspect of UGT1A1 to the aim, otherwise, co-management of HIV and TB is a very broad term.

Methods: This section should be headed as "**Methods and Analysis"** according to the journal guidelines. The methods need to state all the databases that will be searched, so please replace the 'etc' with the actual databases. You can also state how the screening will be done: by two independent reviewers? Remove the keywords here and use the space to give more details on your methods such as how you plan to synthesise your data.

Has the protocol been registered? If so you can also state this here and provide the registration reference

Strength and limitations

The first strength and limitations should state what your rigorous methods clearly are; in this case the use of the JBI methodology and the use of PRISMA-ScR guideline for reporting. You can include "UGT1A1" in the first bullet point.

The objectives should be stated before the methods

Objectives

Line 44: Include the word "summarise" so that it reads; the main objective of this scoping review is to summarise the available evidence etc

For the Methodology, as you list the JBI stages, I recommend that you immediately explain how you will conduct each stage. Its is not necessary to have an initial list of the stages

For example, when identifying the research question you can refer to the PCC framework.

You can then move to the second stage and explain how you plan to identify the relevant studies…

Line 41-60: as stated earlier, the objectives are misplaced here and need to move up before the methodology.

Pg 8 Line 17: There are two "OR"s next to each other. Please remove one

Pg8 Line 31-41: There are over 49 countries in sub Saharan Africa. You can check the United Nations Development Programme site for the complete list. Countries such as Mozambique, are missing on your country list.

Do you plan to conduct a pilot search?

Take your reader through the process step by step. After the database search , where are you going to export your identified studies?

After exporting, how will you de-duplicate the identified articles?

Are you using a particular software for screening?

Page 11: The Prisma diagram 2009 is outdated. You can use the 2020.

https://www.prisma-statement.org/scoping

- As this will be a scoping review, the last text box on your PRISMA should just state the number of included studies in your review. You will not have a meta analysis in a scoping review

Page 11/12 Charting of the data

State explicitly if the data extraction will be conducted by one reviewer and checked by another or if two people will conduct the data extraction independently and then check and discuss any discrepancies until consensus is reached.

The Affiliations can also go to your title page

Include the completed PRISMA_ScR checklist as part of your submission

---

VERSION 1 - AUTHOR RESPONSE

Reviewer: 1
Dr. Qin Jiang, Sichuan Provincial People's Hospital: Sichuan Academy of Medical Sciences and Sichuan People's Hospital
Comments to the Author:
The authors provided many old references, many of them is dated from year 2020. Only one item was since 2020. Thus, the scientific value of this scoping review was restricted by timeliness

Thank you for the opportunity to improve the scoping review protocol. References have been updated to the latest reports and available information. References 1, 2, 3,4,5,9, 10, 15, 16 and 17 are now 2020 and above. References 6–8, 12–14, and 18 predate 2020; however, they are included because they either report foundational scoping reviews, describe earlier studies that identified relevant polymorphisms, or provide definitional sources essential for building the conceptual understanding of the subject. About 71% of people living with HIV in

Southern Africa had transitioned to dolutegravir by December 2023 (Moyo etal, 2025), so we believe the contextual issues being studied are still topical.

Reviewer: 2

Miss Maureen Moyo-Chilufya , Faculty of Health Science University of Pretoria

Comments to the Author:

*** Please find the comments from this reviewer in the attached file ***

Please find my comments in the attachment

Thank you for the opportunity to review the article titled, "**Uridine diphosphate glucuronosyl transferase 1A1 gene polymorphisms and treatment outcomes in HIV and MTB coinfection in sub-Saharan Africa**: **A scoping review protocol**" by Vhanda et al 2026

It is an important topic in the context of Sub -Saharan Africa, which has the highest burden of HIV, globally.

Thank you for the comment

Abstract Introduction: should begin with an introductory statement that explains the association of UGT1A1 with HIV/TB co-infection

Thank you for the comment, the introductory statement has been added, ***lines 32-35***

Line 32 – 35: The aim does not state anything about UGT1A1. The authors should include the aspect of UGT1A1 to the aim, otherwise, co-management of HIV and TB is a very broad term.

Thank you for the comment. The aim statements have been revised in the 2 sections as suggested. ***Lines 42-45, and 89-93.***

Therefore, this scoping review aims to map existing evidence, synthesize findings on how genetic polymorphisms in the UGT1A1 gene affect the metabolism of ARVs and anti-tuberculosis (ATT) drugs, and identify gaps in literature regarding their impacts on drug efficacy, toxicity, and treatment outcomes in SSA.

Methods: This section should be headed as "**Methods and Analysis**" according to the journal guidelines.

Thank you. This has been amended on ***page 5 line 126***

The methods need to state all the databases that will be searched, so please replace the 'etc' with the actual databases. You can also state how the screening will be done: by two independent reviewers? Remove the keywords here and use the space to give more details on your methods such as how you plan to synthesise your data.

Thank you the methods section has been improved as suggested. ***Page 5, lines 129-135***

Data screening is explained on ***Page 7-8, lines 174-181, and on Page 11 on the Prisma diagram****, and plan for data synthesis* ***page 11-12, lines 279-289***

Has the protocol been registered? If so you can also state this here and provide the registration reference

The protocol has been registered on the Open Science Framework on the link. *https://osf.io/nmjq9/overview?view_only=dedb1c00f8ce4d73b7efab125dd99456*

Registration number is still pending

Strength and limitations The first strength and limitations should state what your rigorous methods clearly are; in this case the use of the JBI methodology and the use of PRISMA-ScR guideline for reporting. You can include "UGT1A1" in the first bullet point.

Thank you for the opportunity to improve the article: The first bullet has been amended as suggested. ***Page 2, lines 59-62***

The objectives should be stated before the methods

Objectives

Line 44: Include the word "summarise" so that it reads; the main objective of this scoping review is to summarise the available evidence etc

Thank you. The word 'summarise' has been added. ***Line 116****.*

For the Methodology, as you list the JBI stages, I recommend that you immediately explain how you will conduct each stage. Its is not necessary to have an initial list of the stages

For example, when identifying the research question you can refer to the PCC framework.

You can then move to the second stage and explain how you plan to identify the relevant studies…

Thank you. The initial list of stages has been deleted, only the explained stages remain.

Line 41-60: as stated earlier, the objectives are misplaced here and need to move up before the methodology.

Thank you. The objectives have been moved as suggested. ***Lines 115-125.***

Pg 8 Line 17: There are two "OR"s next to each other.

Thank you. Repeated word was deleted

Please remove one Pg8 Line 31-41:

Thank you, 'one' removed.

There are over 49 countries in sub Saharan Africa. You can check the United Nations Development Programme site for the complete list. Countries such as Mozambique, are missing on your country list.

*Thank you. The missing countries have been added to the list on **page 7***

Do you plan to conduct a pilot search?

Yes, a pilot search will be conducted to refine and validate the search strategy. An initial limited search will be performed in key databases such as PubMed to identify relevant articles and to analyse key terms and Medical Subject Headings (MeSH). These terms will then be used to optimise the full search strategy across all the selected databases. The pilot phase will also help ensure sensitivity and specificity of the search in capturing all relevant studies aligned to the review objectives.

*The pilot search is explained on **Page 10-11 line 259-265***

Take your reader through the process step by step. After the database search , where are you going to export your identified studies?

Following the execution of the final search strategy across all selected databases, all identified records will be exported into a reference management software, specifically Zotero. This will serve as the central repository for managing citations and facilitating subsequent stages of the review process.

*The step-by-step process is summarised on the flow diagram, **page 10, lines 188-200 in Figure 1 (page 11)***

After exporting, how will you de-duplicate the identified articles?

Deduplication will be conducted within Zotero using its automated duplicate detection function, which identifies records based on key bibliographic fields such as title, author(s), and publication year.
Following automated removal, a manual review will be undertaken to ensure that any remaining duplicates not captured by the software are identified and removed, thereby enhancing the accuracy of the dataset.

Page 10 lines 189-194

Are you using a particular software for screening?

The screening process will be managed and documented using Microsoft Excel. Two independent reviewers will screen titles and abstracts against the predefined inclusion and exclusion criteria. The screening will adopt and use standardised codes such as; Include, Exclude and Uncertain. Conflicts will be resolved through discussion, and where necessary, a third reviewer will be consulted. Full-text screening will follow the same procedure to ensure methodological rigour and transparency. ***(Page 8, lines 175-182)***

Page 11: The Prisma diagram 2009 is outdated.

You can use the 2020. https://www.prisma-statement.org/scoping - As this will be a scoping review, the last text box on your PRISMA should just state the number of included studies in your review. You will not have a meta analysis in a scoping review

Thank you, the 2020 version in the provided link has been used to adjust the protocol. Amendments effected

Page 11 Lines 219-254

Page 11/12 Charting of the data

State explicitly if the data extraction will be conducted by one reviewer and checked by another or if two people will conduct the data extraction independently and then check and discuss any discrepancies until consensus is reached.

Thank you. Two independent reviewers will conduct the data extraction independently and then checked and any discrepancies discussed until a consensus is reached. ***Lines 176-182***

The Affiliations can also go to your title page

Thank you. Affiliations have been moved to the title page. ***Lines 7-24***

Include the completed PRISMA_ScR checklist as part of your submission

Thank you. The PRISMA_ScR checklist has been included
